# The role of the European Union in health policies of member states – an example of the rare diseases policy in Croatia

**DOI:** 10.3325/cmj.2021.62.553

**Published:** 2021-12

**Authors:** Snježana Ivčić, Dagmar Radin

**Affiliations:** 1 Croatian Academy of Sciences and Arts, Zagreb, Croatia; 2 Faculty of Political Science, University of Zagreb, Zagreb, Croatia

## Abstract

**Aim:**

To assess the European Union's (EU) impact on the Croatian health policy, and identify which mechanisms and processes were used to shape a particular health policy on the EU and national levels. The study focused on the rare diseases policy to obtain a better insight into the process of policy shaping, starting at the EU level and moving down to the Croatian national level.

**Methods:**

We conducted actor analysis, policy networks, and semi-structured qualitative interviews with key policy actors at the EU and domestic level. The analysis of actors included actor mapping, the analysis of their relationships, and of their interdependence. Policy networks involved identifying key actors and analyzing them separately to create both policy networks to explain their hierarchy and relationships. Semi-structured interviews included ten key experts at the EU and national health policy levels.

**Results:**

The implementation of the EU health policy is complex. Hard and soft law were complementary in the way they affected the translation of EU rare diseases policy into Croatian law. Strong and interconnected EU and domestic actors were significant in this process, which resulted in the creation of Croatia’s rare diseases policy.

**Conclusion:**

Given that the rare diseases policy area is a developing policy area, this study contributes to a better understanding of the implementation of the EU health policy, clarifying a mechanism that can enable national governments to adopt specific health policies.

Health policy, compared with other sector policies, is a latecomer to the European Union’s (EU) agenda. The EU health policy is first mentioned in Article 129 of the Maastricht Treaty (1992), followed by the Amsterdam Treaty (1997). In 2007, the Lisbon Treaty (Article 168) ensured a great level of human health protection while stating that the EU would respect the competence of member states in defining their health policies, in health care organization, and in the provision of health services. Even though member states formally have full responsibility for their respective health policies, EU's competence in the field of health care has extended over time (1). Specifically, following the 2008 economic crisis, the EU laid out the foundation for the new control mechanisms for member state spending/budgeting, which has since strongly affected health policies. The new control mechanisms, known as new economic governance, include a set of regulations and procedures adopted during the 2008 economic crisis and implemented during the initial stages of the European semester in 2010. The country-specific recommendations introduced in 2012 were specifically directed to member states, which affected the Croatian health care system as well.

The rare diseases policy provides a good example for the analysis of the EU’s impact on shaping the health policies of member states for several reasons: the complexity of diagnoses falling into this definition, the associated high-cost burden of treatment, and the spillover effect that rare diseases have on the patients’ family and other social and economic burdens surrounding the care of the patient. “Between 27 and 36 million people in the EU are affected by rare diseases, representing 6-8% of the population, with estimated 5 to 8 thousand different diagnoses” (2), thus posing an important public health problem. Rare diseases, defined as illnesses occurring in fewer than five individuals per 10 000 of the population (3), are specific for several reasons, and “patients suffering from rare diseases have traditionally been marginalized due to several factors including: limited scientific research on illnesses, a small number of patients, lack of medical expertise, little public awareness, and a small number of medicines available” (4). In Croatia, about 250 thousand people are affected by rare diseases (5), but accurate and comprehensive epidemiological data are not available. Additionally, lack of proper diagnosis and integrated care makes it even more difficult for identification and monitoring purposes. Rare diseases officially entered the Croatian public space in 2002 with the establishment of the Rare Diseases Croatia (*Hrvatski savez za rijetke bolesti*).

Rare diseases were chosen over other areas (eg, HIV, cancer, and heart disease) because of the scarcity of related policy research as compared with other diseases. Furthermore, the rare diseases policy is created differently than other health policies in the EU because they affect fewer patients compared with other diseases. Another reason are related high drug costs, which do not stimulate the interest of the pharmaceutical industry to develop drugs without dedicated support from state institutions. To that end, the EU took some steps to improve the situation by adopting the Orphan Regulation No 141/2000 in 1999 to encourage the pharmaceutical industry to develop expensive drugs necessary for the treatment of rare diseases. Furthermore, the establishment of the European Medicines Agency (EMA) in 1995, and the EUROPLAN in 2008 were steps toward the creation of preconditions for the process of Europeanization of the rare diseases policy. Directive 2011/24/EU, or more specifically, the Cross Border Directive, was adopted in 2011, allowing EU citizens to seek health care in another EU or EEA member state, which facilitated seeking expensive and specialized care for patients with a rare disease, as well as making specific reference to rare diseases (articles 54 and 55).

In this article, we developed a theoretical model explaining the impact of Europeanization and neofunctionalism on national health policies and what they have in common (6). The theoretical framework (Figure 1) defines the process of Europeanization and neofunctionalism on EU health policies in general, that is, the influence of the EU on the national health policies of member states in particular. The compatibility of these two theoretical perspectives clarifies how the health policy implementation process at the EU level affects member states (6), particularly in the context of rare diseases policy.

**Figure 1:**
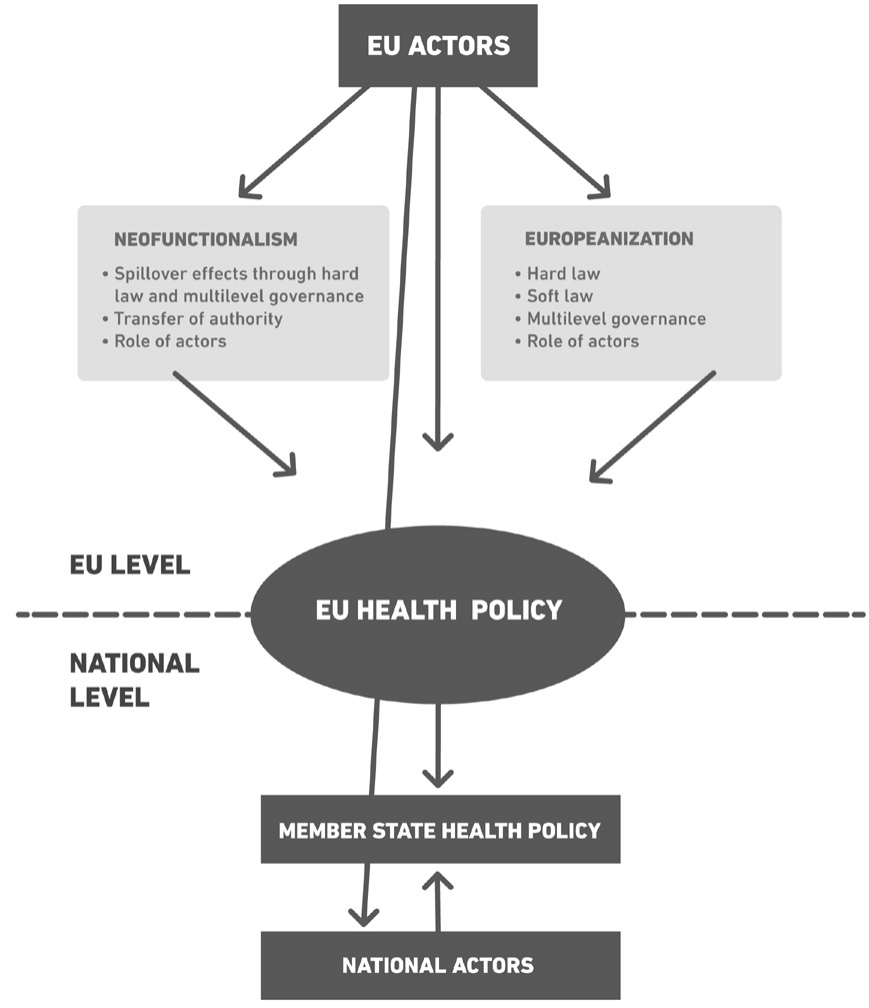
A theoretical model showing the impact of the concept of Europeanization and the theory of neofunctionalism on European Union and national health policies.

In this study, we define Europeanization as multilevel governance (7-9), and it includes the influence of national actors that strive to achieve their goals and influence health policy shaping at the national level with the assistance of EU institutions and supranational agencies (10). This definition, in some areas, coincides with the neofunctionalist interpretation of the spillover effects through multilevel governance (11). Health policies are moderately shifted into Europeanization, although most member states refused this concept and recognize health policy a national issue (12).

The concept of neofunctionalism includes the spillover effect, transfer of authority, the role of actors, and the influence on multilevel governance (11,13,14); all aspects that are found in the EU health policy formulation and that can help explain its implementation. For example, because of the spillover effect, health policies have come under the influence of market policies (15), whose influence is also visible in the transfer of authority (through supranational agencies), the role of actors, a spillover of DGS' responsibilities (DG Health and Food Safety, DG Internal Market, Industry, Entrepreneurship and SMEs, DG Employment, Social Affairs and Inclusion), and the crucial role of the European Court of Justice (ECJ) of the EU.

The EU uses hard and soft law successfully to achieve its goals by shaping health policies, and the rare disease policy is not the first example of a health policy under the EU influence. The hard law mechanism refers to the decisions of the ECJ and to EU regulations (16) that are absolute in their authority and directly applicable to all member states. Such directives leave to the national authorities the choice of form and method but demand that each member state manages defined results. Soft law refers to the rules of conduct (declarations, conclusions, harmonization through recommendation) that are not legally enforceable, but nonetheless have a legal scope in that they guide the conduct of institutions, member states, and other policy participants (17,18).

This article explored various instruments and mechanisms that the EU uses to shape and transfer policies to the national level. The aim was to answer the main research questions: What is the EU's impact on the Croatian health policy, and what mechanisms and processes were used to shape a particular health policy on the EU and national levels? The study focused on the rare diseases policy to obtain a greater insight into the process of policy shaping, starting at the EU level and moving down to the Croatian national level.

## Methods

This study used several qualitative methods, including actor analysis, policy networks, and semi-structured qualitative interviews with key actors at the EU and national level.

The analysis of actors involved in public policy shaping included actor mapping, the analysis of their relationships, and of their interdependence (19). Networks proved to be a better way of connecting in the field of health care than the Open Method of Coordination (15), which is why they were included in this study. Furthermore, they have a greater influence on particular policies, notably in relatively marginalized policy areas that are not part of high politics nor do they involve high expenses. An analysis of policy networks (20) shows how actors alternate resources to be more effective and how they mutually make changes and interact at the both EU and national policy levels. We accomplished the analysis by identifying key actors and analyzing them separately to create/discover both policy networks and explain their hierarchy and relationships (Figure 1).

The ten participants selected for the interviews were key actors responsible for the shaping of rare disease policies at the both the EU and domestic level. They were rare disease experts at the EU and the domestic level: three representatives of EURORDIS Rare Diseases Europe, the DG Health and Food Safety, and the European Medicine Agency (EMA) respectively; and seven domestic actors, representatives of state bodies, professional societies, medical institutions, patient associations, and the pharmaceutical industry (6). Most of the selected experts were from Croatia. The semi-structured interviews were conducted from March 30, 2016, to April 1, 2017. All the interviewees were asked the same questions, although not necessarily in the same order. The range of responses varied depending on the individual's area of expertise, and the interviews lasted from 40 to 60 minutes.

A semi-structured interview generally allows a deeper and better insight into a process. In addition, topics not mentioned in official documents can be discussed, and participants can be more easily approached (21). A special type of semi-structured qualitative interviews is the interview with experts (22), which was applied in this study. Since experts are not average interlocutors, and for the purpose of this type of interview, it is important that the chosen experts are recognized in their field (23).

Two criteria were used in actor mapping: positional (formal position of the actor in a decision-making process) and reputational (key information about the actor is given by relevant insiders) (24). The formal position of the actors was assessed based on public data, whereas the reputational position was based on the data gathered during the semi-structured interviews. The application of these two criteria resulted in a list of the key actors, their interests, and resources.

All the experts were asked about 18 different topics (Table 1), and their answers differed according to their field of expertise. The answers were analyzed through qualitative methods, and helped to recreate relations between EU and national actors and explain the resources they use to achieve their goals.

**Table 1:** Interview questions/areas*

1. The role of state institutions (MZ, HZZO, HZJZ, HALMED)
2. The role of public health/medical institutions
3. The role of professional medical associations
4. Clinical trials of rare diseases
5. Scientific projects addressing rare diseases
6. Putting drugs on the national list of drugs/orphan drug problem
7. Drug approval (EMA, HALMED)/orphan drug problem
8. Development of the Register of Rare Diseases
9. The role of the pharmaceutical industry
10. Rights of patients with rare diseases
11. Financing/financial aspects generally related to rare diseases
12. The role of EU actors (DGs, EMA, expert groups)
13. Cooperation with EU actors at all levels
14. Cooperation between different actors
15. Orphanet (a portal for rare diseases and orphan drugs)
16. Preventive activities (eg, neonatal screening)
17. The role of patient associations at national and EU levels
18. Raising public awareness about rare diseases

*Abbreviations: MZ – Ministry of Health; HZZO – Croatian Health Insurance Fund; HZJZ – Croatian Institute of Public Health; HALMED – Agency for Medicinal Products and Medical Devices of Croatia; EMA – European Medicines Agency; DG – Directorate General; EU – European Union.

**Figure 2:**
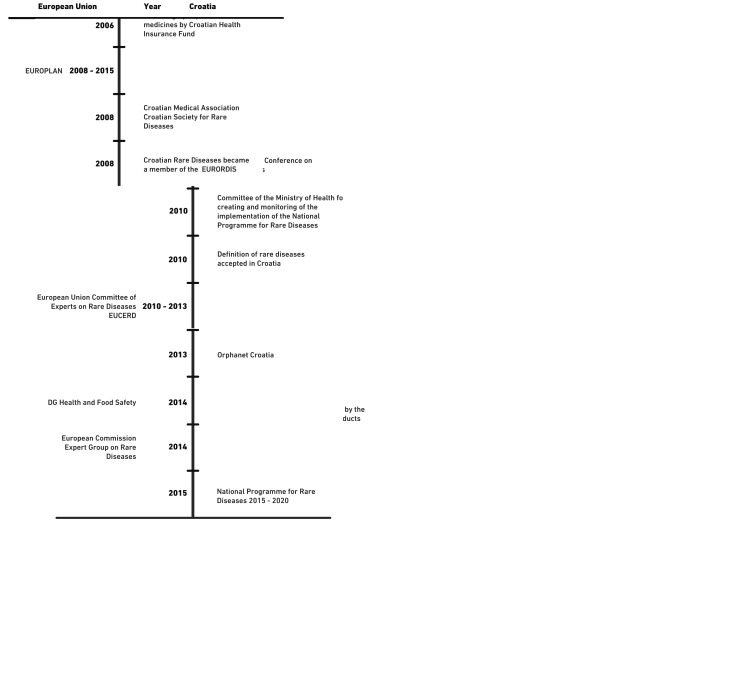
Timeline of key events in development of the rare diseases policy at the European Union (EU) and national levels.

Figure 2 outlines the basic timeline, events, and developments of the rare disease policy at the EU and domestic levels, showing the influence of the EU, as well as the overlapping of events during the process of shaping the rare diseases policy in Croatia.

## Results

Certain mechanisms (such as hard and soft law) are complementary in the way they affect the desired goals with the help of national and EU actors (6).

**Figure 3:**
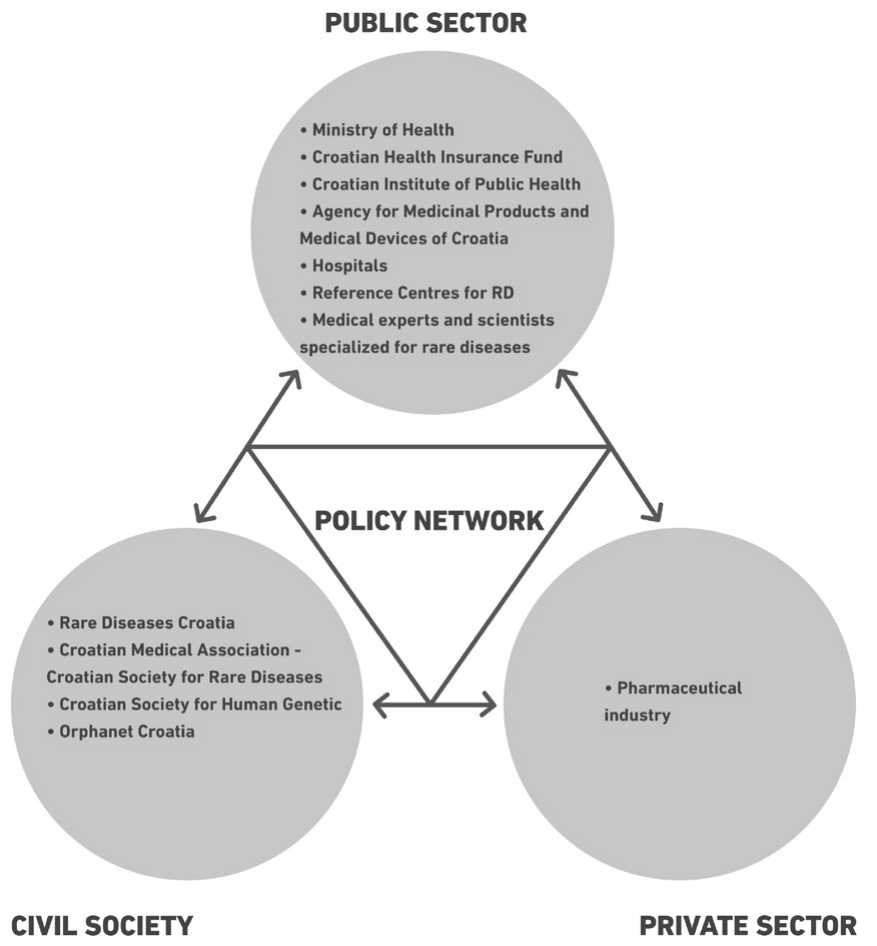
Policy network at the national level (eg, Croatia). DG – directorate general.

**Figure 4:**
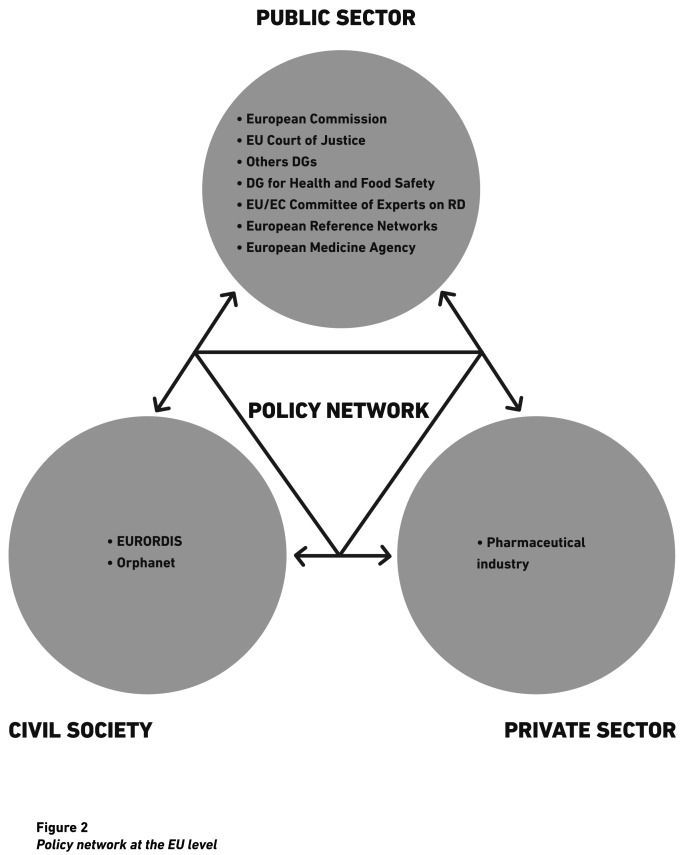
Policy network at the European Union (EU) level. DG – directorate general; EC – European Commission; RD – research and development.

More specifically, state and non-state actors at the domestic and EU level are intertwined in shaping the rare disease policy and in the way they transfer it from the EU to the domestic level (Figure 3 and 4). There are interconnections among the actors at both levels. Networks in the rare diseases policy allow for changes to be more concrete and they can mobilize greater support because of their focus on a very narrow area.

The policy network at the national level in Croatia is composed of domestic actors and their mutual connections. In addition to the Ministry of Health (MoH), which holds the most important formal position, other actors also play a significant role in shaping and transferring the policy from the EU level. Although the MoH has the legal (and political) power to shape policies, a very strong actor in this process is Rare Diseases Croatia, a nonprofit NGO. This so-called *spiritus movens* links all the other actors because of its great influence in the public sphere. Health institutions and professional associations represent the professional influence, and the pharmaceutical industry provides financial resources. To achieve their goal, these actors worked together to create the National Programme for Rare Diseases 2015-2020 (National Programme).

The results also show the way in which the European actors (ECJ, EMA, DGs, EURORDIS, etc) use hard and soft law to achieve their goals, as illustrated in the timeline (Figure 2) and theoretical model (Figure 1).

As expected, the presence of stronger domestic actors brings about positive legal changes. More specifically, the EU uses hard and soft law with the help of the ECJ, regardless of each member state’s actual needs or organizational and financial resources. A soft law can emerge into a hard law, as evidenced by the fact that many member states officially adopted national programs or plans for rare diseases, which soon became part of their official health policy. An example of this is that, in the framework of the EUROPLAN 2008-2015, funded by the EC, some member states, including Croatia, adopted plans or programs for rare diseases. In Croatia, there was a recognized basic need for a rare diseases policy, but it was the help of the EU actors and their collaboration with the domestic actors in the professional, political, and civic field (Figure 3 and 4) that ensured a positive result in the creation of a national rare diseases policy. In fact, while the policy entered the Croatian public space in 2002, the National Programme was adopted only in 2015, after Croatia's participation in EUROPLAN, as shown in the timeline (Figure 2) outlining the parallel development of the rare diseases policy at the EU and domestic levels. Here, it is important to understand how the policy network at the EU level interconnects the relevant actors and enables them to share information and exchange resources in order to reach the desired policy goals. While the European Commission is at the top of the policy process, followed by the EU Court of Justice and the EMA, other actors strongly participate in the process of shaping and transferring the policy at the EU level. More specifically, while the EU institutions have the legal and political power, the EURORDIS (representing patients) and the pharmaceutical industry are also very strong and influential actors because of their presence and influence in the public sphere and their financial resources. This was mostly evident in the adoption of the Orphan Regulation (EC) No 141/2000 in 1999, which encouraged the pharmaceutical industry to develop expensive drugs necessary for treating rare diseases. The collaboration with the pharmaceutical industry is not always obvious and transparent, although its existence and extent can be indicated though available information online provided by civil society organizations, including donations and sponsorships. However, the impact of the pharmaceutical industry is more significant in that the industry also collaborates with state institutions engaged in medicines and health policies. Hard data for this are elusive, except for the information that can be obtained through the interviews (eg, in Croatia there is a strong interest in establishing a database of people with rare diseases).

The results from the semi-structured interviews showed that actors had different perspectives on the shaping of the rare diseases policy, which depend on their interests and resources. The interviews also shed new light on the relations between actors in terms of how they interact and how they influenced the shaping of the rare disease policy either in Croatia or at the EU level. This proved to be very valuable for learning about the processes taking place at the national level, particularly in Croatia, where research on this new policy is scarce and difficult to find.

## Discussion

The case of Croatia shows the role that domestic and European actors played in the process of transferring the rare diseases policy from the EU to the national policy level. By acting horizontally and vertically, and through various mechanisms of hard and soft law (spillover effect, the transfer of authority, the role of actors, and the influence on multilevel governance), they successfully imposed policies on the member state. While this shaping of the rare diseases policy was in part based on the adoption of legal regulations that could be imposed on member states, a large part of the “imposition” took place through informal mechanisms, including soft law that can, and often does, evolve into hard law. Supranational agencies, such as the EMA, also play a part in this process, considering that they have been given authority and more power in the decision-making process than member states, which are expected to abide by the EU's decisions. In Croatia, domestic actors, with the help of soft law methods and European actors (who helped them strengthen their capacities), were able to include the rare diseases policy on the agenda with the aim of making legal changes. As we anticipated, hard and soft law did increase the possibilities for (positive) legal changes. We also anticipated that stronger domestic actors would advance more (positive) legal changes. In both cases, the results matched our expectations and supported our hypothesis.

The 1999 adoption of the Orphan Regulation, along with the establishment of the EMA, and the decisions of the ECJ have created the basis for the implementation of hard law in member states, although the more subtle soft law has had a farther-reaching impact (6). This significant influence of soft law and of policy actors is visible in the shaping of the rare diseases policy in Croatia. As a result, Rare Diseases Croatia was established in 2002; the Croatian Medical Association founded the Croatian Society for Rare Diseases in 2008; agreement was reached on the definition of rare diseases at the domestic level in 2010; and the National Programme for Rare Diseases 2015-2020 was adopted in 2015. Strong actors at the EU and domestic levels proved to be crucial for the implementation, notwithstanding the independent influence of the hard and soft law. Insights from interviews with experts who are insiders to the process allowed us to connect how their interests and resources work together and intertwine. Furthermore, as publicly available data and other studies about shaping health policies (in general) in Croatia are rare, reconstructing how one policy is shaped from its inception is difficult without the insight from expert semi-structured interviews. Combining several qualitative research methods enabled us to analyze the complex role of the actors at the EU and national level and how policy networks are designed given their central role in shaping the rare diseases policy.

The results of the study show the resulting National Programme for Rare Diseases as a good example of how soft law can evolve into hard law (at the national level), facilitated by the significant involvement of policy actors. In conclusion, this study finds the theory of neofunctionalism and the concept of Europeanization appropriate to explain the mechanisms through which EU actors affect the shaping of national health policies. The example of the rare diseases policy shows the complementary nature of soft and hard law, and their influence in the creation of the national policy of rare diseases.
